# Genetic Diversity, Occult Hepatitis B, and Mutational Signatures in Migrants from Regions with Varying HBV Endemicity: Importation of Diverse Viral Variants into St. Petersburg, Russia

**DOI:** 10.3390/ijms27136065

**Published:** 2026-07-06

**Authors:** Elena N. Serikova, Yulia V. Ostankova, Alexandr N. Shchemelev, Nadezhda A. Pechnikova, Edward S. Ramsay, Areg A. Totolian

**Affiliations:** Saint Petersburg Pasteur Institute, 14, ul. Mira, Saint-Petersburg 197101, Russia; genista.bio@gmail.com (E.N.S.); shenna1@yandex.ru (Y.V.O.); nikanobelevka@gmail.com (N.A.P.); totolian@spbraaci.ru (A.A.T.)

**Keywords:** hepatitis B virus (HBV), occult hepatitis B infection (OBI), migrants, nested real-time PCR, molecular diagnostics, genotyping, S-gene mutations

## Abstract

The importation of hepatitis B virus (HBV) variants through migration challenges elimination efforts in low-endemicity countries. This study evaluated the seroprevalence, occult hepatitis B infection (OBI), and molecular signatures of HBV among 537 international migrants from 46 countries who arrived in St. Petersburg, Russia. HBsAg, anti-HBs, and anti-HBc were measured by ELISA. This was followed by nested real-time PCR targeting viral genes (S, X) and a human housekeeping gene (HPRT, internal control), amplification of overlapping fragments covering the complete HBV genome, and Sanger sequencing. HBsAg prevalence was 2.61% (95% CI: 1.43–4.34), while anti-HBc was detected in 16.39% (95% CI: 13.36–19.79). HBV DNA was found in 8.19% (44/537; 95% CI: 6.02–10.04) of migrants. Notably, OBI (HBsAg-negative/HBV DNA-positive) was identified in 33 individuals, yielding a prevalence of 6.15% (33/537; 95% CI: 4.27–8.52) in the entire cohort and 6.31% among HBsAg-negative subjects. The findings among OBI cases were as follows: the majority (54.5%) had no detectable anti-HBc or anti-HBs; 30.3% were positive for anti-HBs only; 6.06% were positive for anti-HBc IgG only; 9.09% of cases featured both anti-HBc and anti-HBs. Viral loads in OBI cases were uniformly low (14–53 IU/mL). Genotype D predominated (86.36%, 38/44). The distribution of subgenotypes among all sequenced isolates was as follows: D1 in 36.36% (16/44), D2 in 36.36% (16/44), D3 in 13.64% (6/44), while genotypes A2 (6.82%, 3/44), B4 (4.55%, 2/44), and C2 (2.27%, 1/44) were rare. In the major hydrophilic region (MHR), the most frequent amino acid substitutions were at positions T127P/I (61.4%) and T118R/M/V/A (45.5%). Immune escape mutations were significantly associated with OBI (81.82% of OBI cases versus 18.18% of HBsAg-positive cases; *p* = 0.003). Drug resistance mutations (L180M + M204V ± T184A) were detected in 11.4% of isolates (all genotype D2). In the precore region, stop codon mutation W28* was found in 31.82% of samples, and the A1762T/G1764A double substitution in the basal core promoter occurred in 25.0%. The high prevalence of OBI, coupled with the accumulation of escape and drug resistance mutations in imported HBV variants, highlights the urgent need to include molecular HBV screening in mandatory medical examinations for arriving migrants to prevent undetected transmission and inform clinical management in receiving countries.

## 1. Introduction

Hepatitis B virus (HBV) infection remains a major global public health challenge, despite the availability of effective vaccines and antiviral therapies. According to the World Health Organization (WHO), an estimated 254 million people were living with chronic HBV infection in 2022, and the virus accounted for approximately 1.1 million deaths annually, primarily due to cirrhosis and hepatocellular carcinoma [1]. Although global vaccination efforts have significantly reduced the incidence of new infections, many countries remain off track to meet the 2030 elimination targets set by the WHO [2].

HBV exhibits remarkable genetic diversity, with ten recognized genotypes (A–J) that display distinct geographical distributions and clinical implications [3,4,5]. Genotype D is predominant in the Mediterranean region, the Middle East, and parts of Eastern Europe, including Russia, while genotype A is more common in Northwestern Europe and sub-Saharan Africa. Genotypes B and C prevail in East Asia, and genotype E is concentrated in West Africa [6]. This geographical structuring reflects historical patterns of viral evolution and human migration over millennia.

International migration has dramatically reshaped the global distribution of infectious diseases, including HBV. Migrants often originate from regions with moderate to high HBV endemicity and move to countries with lower prevalence, potentially introducing diverse viral variants into recipient populations [7,8,9]. The scale of this phenomenon is substantial: in the European Union/European Economic Area, migrants accounted for 34% of all new HBV diagnoses in 2023, with prevalence rates among migrant populations reaching up to 31.7% depending on the country of origin [10]. Systematic reviews confirm that the prevalence of blood-borne viruses among newly arrived migrants in Europe can be as high as 8.7%, underscoring the urgent need for community-based screening programs [11]. Similarly, among refugees and displaced persons in low- and middle-income countries, HBV prevalence varies from 1% to 60%, driven by factors such as unsafe medical procedures, low vaccination coverage, and barriers to healthcare access [12].

Beyond overt chronic infection, occult hepatitis B infection (OBI) represents a particularly challenging clinical and epidemiological entity. OBI is defined by the presence of HBV DNA in the liver or serum of individuals testing negative for hepatitis B surface antigen (HBsAg), with or without antibodies against hepatitis B core antigen (anti-HBc) [13,14]. The global prevalence of OBI varies considerably across populations, with higher rates observed in high-risk groups such as individuals with HIV coinfection, hemodialysis patients, and migrants from endemic areas [15,16,17]. OBI retains the capacity for viral reactivation under immunosuppressive conditions [18,19,20], may contribute to hepatocarcinogenesis [21], and represents a potential risk for HBV transmission through blood transfusion or organ transplantation [22,23].

The molecular mechanisms underlying OBI often involve mutations in the S gene, particularly within the immunodominant region encoding HBsAg, which can alter antigenicity and compromise detection by commercial immunoassays. Additionally, mutations in the pre-core and basal core promoter regions may reduce HBeAg expression, facilitating immune evasion [14]. The detection of OBI therefore requires highly sensitive molecular methods because viral loads are typically very low.

Migrants from high-endemicity regions may harbor not only overt HBV infection, but also OBI at significant rates. Several studies have documented that a considerable proportion of HBsAg-negative migrants remain positive for anti-HBc and carry detectable HBV DNA, indicating occult infection [24,25]. These individuals often remain undiagnosed during routine screening if only HBsAg is tested, posing a hidden risk for reactivation upon immigration-related stress, immunosuppressive therapies, or pregnancy. Furthermore, migrants may carry HBV genotypes or variants that are uncommon in the host country, including vaccine escape mutants or strains associated with more rapid disease progression [26,27].

The importation of diverse HBV variants into low-endemicity countries through migration has important public health implications. It may alter the local molecular epidemiology of HBV, complicate clinical management due to genotype-specific differences in disease outcomes and treatment responses, and potentially introduce strains that evade diagnosis or vaccination [28,29,30]. Understanding the genetic diversity, prevalence of OBI, and mutation signatures in migrant populations is therefore critical for informed screening policies and clinical practice.

The Russian Federation, particularly its major cities like St. Petersburg, has experienced increasing migration flows from regions with intermediate to high HBV endemicity, including Central Asia, the Transcaucasus, and Eastern Europe [31,32,33]. Studies have shown that the seroprevalence of HBsAg among migrants in Russia significantly exceeds that in the general Russian population [32]. However, in the Russian Federation, migrants are legally required to undergo testing for HIV, tuberculosis, leprosy, and syphilis, but not for viral hepatitis B or C, despite these infections being officially classified as posing a danger to others. This regulatory gap, combined with the feminization of migration flows and the high proportion of female migrants of reproductive age from endemic regions, creates a potential for undetected HBV importation and transmission, including vertical transmission [34,35,36]. Furthermore, research on infectious diseases among migrants in Russia remains limited compared to other high-income receiving countries [34], underscoring the need for molecular epidemiological studies in this population.

This study aimed to evaluate the prevalence of hepatitis B serological markers, including occult hepatitis B infection detected by a high-sensitive nested real-time PCR assay, and to determine the genetic characteristics of the identified HBV isolates among migrants in St. Petersburg, Russia.

## 2. Results

### 2.1. Study Design

**Figure 1:**
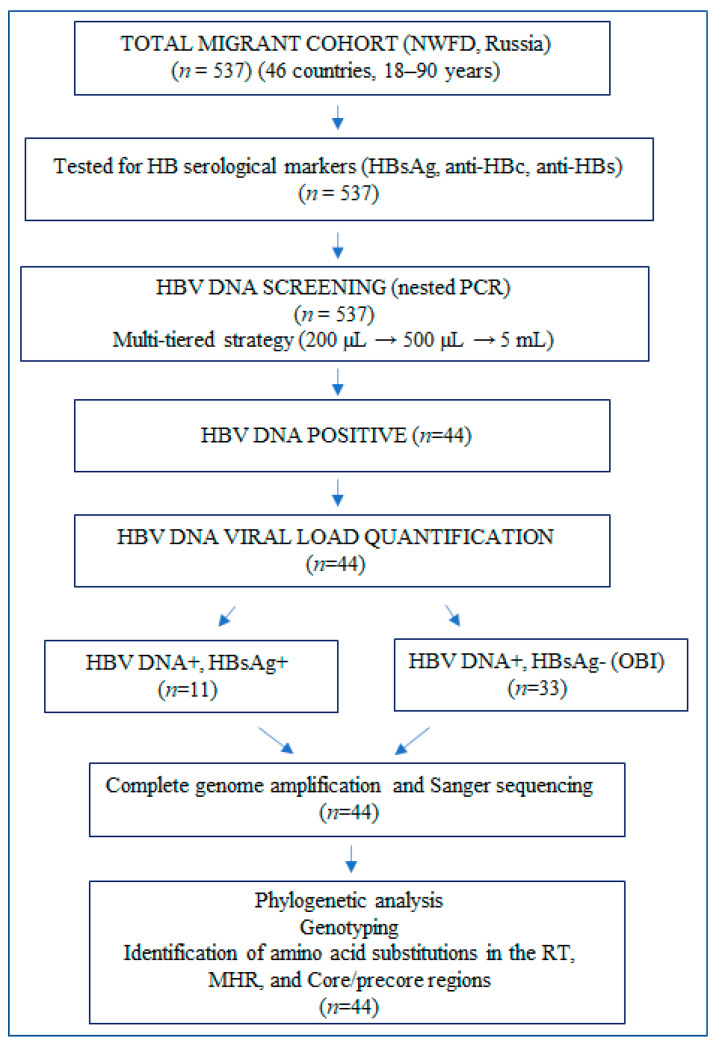
Flowchart of laboratory analysis performed on study samples.

The study population consisted of 537 international migrants residing in the Northwestern Federal District of Russia. The selection of participant samples for each type of analysis (workflow) is presented visually in Figure 1.

### 2.2. Seroprevalence of HB Markers

Among the entire cohort (*n* = 537), HBsAg was detected in 14 individuals (2.61%), anti-HBc IgG in 88 (16.39%), and anti-HBs IgG in 144 (26.82%). The distribution of HB serological profiles (HBsAg/anti-HBc IgG/anti-HBs IgG) in the studied cohort allows the tentative differentiation among vaccine-induced immunity, past resolved infection, and current active infection. A total of 97 study participants (18.06%) showed a serological pattern characterized by the presence of anti-HBs alone. This finding, while most consistent with immunity acquired through vaccination, can also emerge among individuals who have successfully cleared an earlier infection and subsequently lost their anti-HBc IgG reactivity over an extended period. In another 29 subjects (5.4%), only anti-HBc IgG was detectable. Such an isolated anti-HBc IgG pattern raises two possibilities: either a resolved infection with subsequent disappearance of anti-HBs IgG, or an occult HBV carrier state. Serological evidence pointing to a fully resolved natural hepatitis B infection, meaning concurrent positivity for both anti-HBc IgG and anti-HBs IgG, was documented in 47 cases (8.75%). The results of the analysis of the prevalence and distribution of the studied serological markers of hepatitis B in the group are presented in Table 1.

**Figure A1:**
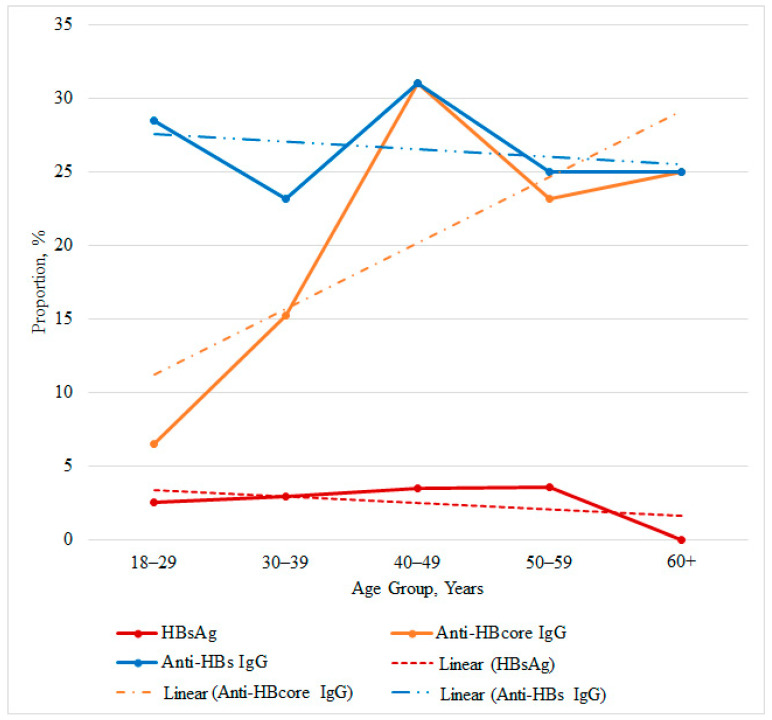
Spearman rank correlation between age groups and prevalence of HBsAg, anti-HBc IgG, and anti-HBs IgG. Points represent observed prevalence for each age group; lines connecting points indicate changes between consecutive age groups. Linear trends fitted by the least-squares method are shown for visualization purposes only and do not represent a separate regression analysis. Spearman correlation coefficients (rs) and two-sided *p*-values are as follows: for HBsAg, rs = 1.735 × 10^−19^, *p* > 0.9999; for anti-HBs, rs = −0.1539, *p* = 0.7833; for anti-HBc, rs = 0.7, *p* = 0.2333. Numerical data are provided in Table A2.

Evaluation of marker prevalence according to sex revealed no differences between males and females (Appendix A Table A1). No significant age-related trend was detected for HBsAg, anti-HBc IgG, or anti-HBs IgG prevalence. However, for anti-HBc IgG, a marked age-related increase was observed, rising from 6.5% in individuals aged 18–29 years to 31.03% in those aged 40–49 years (χ^2^ = 28.41, df = 1, *p* < 0.0001). For detailed data, see Figure A1 and Table A2 in the Appendix A.

Among participants positive for each of the three markers, we assessed the contribution of migrants from different countries of origin. A total of 7 countries contributed to HBsAg seropositivity (*n* = 14 positive cases). The largest contributions came from Uzbekistan (28.6%), Moldova (21.4%), and Tajikistan (21.4%). For anti-HBc IgG (*n* = 144 positive cases), positive cases originated from 28 countries, with the greatest contributions from Uzbekistan (18.1%), Kazakhstan (18.1%), and Ukraine (13.2%). For anti-HBs IgG (*n* = 88 positive cases), positive cases came from 19 countries, with Uzbekistan (21.6%) and Ukraine (21.6%) showing the highest contributions, followed by Tajikistan (11.4%). However, due to the small number of cases in most countries, the confidence intervals were wide and overlapping, and no statistically significant differences between countries were detected. Detailed country-specific data are presented in Appendix A Table A3, Table A4 and Table A5.

### 2.3. HBV DNA Prevalence, Viral Load, and Occult Infection

Among the examined migrants, HBV DNA was detected in 44 individuals, representing 8.19% (6.02–10.04). The highest prevalence was observed in the 50–59 year age group (30.36%, 17/56), which was significantly higher than in participants aged 18–49 years (8.94%, 38/425; χ^2^ = 20.34, df = 1, *p* < 0.0001). In contrast, only 2 out of 56 individuals (3.57%) aged 60 years and older tested positive for HBV DNA, a rate significantly lower than that in the 50–59 year group (*p* = 0.001). No other significant differences between age groups were detected. Detailed age-stratified data are presented in Table 2.

The prevalence of HBV DNA did not differ significantly between males and females. Among males, 25 out of 273 (9.16%, 95% CI: 6.01–13.22) tested positive, compared to 19 out of 264 females (7.20%, 95% CI: 4.39–11.01) (*p* > 0.05).

Among participants positive for HBV DNA (*n* = 44), we assessed the contribution of migrants from different countries of origin. Positive cases originated from 14 countries. The largest contribution came from Uzbekistan, accounting for 11 out of 44 cases (25.0%, 95% CI: 13.19–40.34). Other notable contributions were observed from Belarus (13.6%, 6/44), Kazakhstan (11.4%, 5/44), and Ukraine (9.1%, 4/44). The remaining 10 countries each contributed fewer than 5% of cases. Detailed country-specific data are presented in Appendix A Table A6.

While the above analysis shows the distribution of positive cases, it does not account for differences in the number of migrants originating from each country. Therefore, we calculated the prevalence of HBV DNA among migrants from each country that had at least one detected positive case. The prevalence varied considerably across countries, ranging from 3.01% (Ukraine) to 75.0% (Vietnam). However, the very small number of migrants from several countries (e.g., Vietnam, Lithuania, Latvia) resulted in wide and overlapping confidence intervals, and no statistically significant differences between countries could be reliably demonstrated. Detailed data are presented in Appendix A Table A7.

Viral load was measured in all 44 migrants with detectable HBV DNA. Of the 14 HBsAg-positive individuals in the cohort, 11 had detectable HBV DNA; the remaining three were HBsAg-positive but HBV DNA-negative. Two distinct patterns were observed based on HBsAg status. Among the 11 HBsAg-positive individuals with detectable HBV DNA, viral load ranged from 157 IU/mL to 6,250,000 IU/mL. Nine of these participants had viral loads between 1200 IU/mL and 6,250,000 IU/mL (mean ~938,000 IU/mL, median 115,000 IU/mL), while the remaining two had low viral loads of 157 IU/mL and 214 IU/mL, respectively. In contrast, among the 33 individuals who tested negative for HBsAg, viral load was uniformly low, ranging from 14 IU/mL to 53 IU/mL (mean 33.2 IU/mL, median 33 IU/mL). Thus, OBI was identified in 33 migrants, corresponding to a prevalence of 6.15% (33/537, 95% CI: 4.27–8.52) in the overall cohort and 6.31% (33/523, 95% CI: 4.38–8.75) among HBsAg-negative individuals.

Among the 33 individuals with occult hepatitis B infection (HBsAg-negative, HBV DNA-positive), we examined the distribution of anti-HBc IgG and anti-HBs IgG. The majority (18/33, 54.5%, 95% CI: 36.35–71.89) had no detectable anti-HBc or anti-HBs. Only anti-HBs IgG was present in 10 cases (30.3%, 95% CI: 15.59–48.71), while only anti-HBc IgG was found in only 2 cases (6.06%, 95% CI: 0.74–20.23). Concurrent positivity for both anti-HBc and anti-HBs was observed in 3 cases (9.09%, 95% CI: 1.92–24.33).

### 2.4. HBV Genotyping

**Figure 2:**
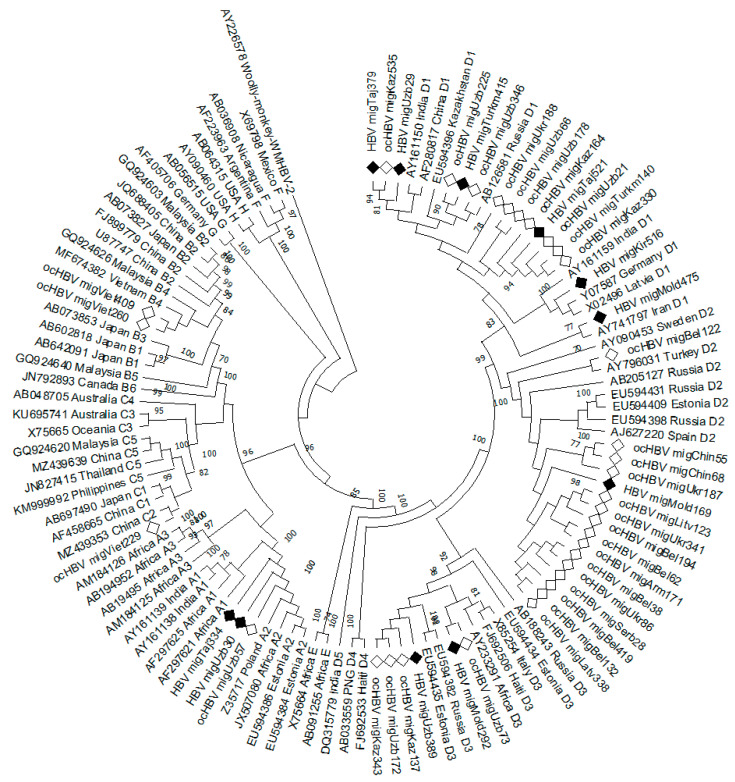
Phylogenetic analysis of HBV whole-genome nucleotide sequences isolated from migrants. The tree was constructed using the neighbor-joining (NJ) method based on the Kimura 2-parameter (K2P) model with 1000 bootstrap replicates. Bootstrap values ≥ 70% are shown at the nodes. Reference sequences available in GenBank were used for comparison and are designated with accession numbers indicating genotype and region of sample origin. The Woolly Monkey HBV nucleotide sequence (AY226578) was used as the outer group. The samples studied in this work are indicated by black diamonds (HBsAg+) and white diamonds (HBsAg−).

The whole-genome nucleotide sequences of identified HBV isolates have been submitted in GenBank under accession numbers PZ544518-PZ544561. Phylogenetic analysis was performed on the obtained nucleotide sequences (Figure 2).

A total of 44 migrants were enrolled in the molecular genetic analysis. The dominant genotype was D, detected in 38 individuals (86.36%, 95% CI: 72.6–948). Within this genotype, three subgenotypes were resolved: D1 and D2 were equally frequent (*n* = 16 each, 42.11% of D isolates, 95% CI: 26.3–59.2), followed by D3 (*n* = 6, 15.79%, 95% CI: 6.0–31.3). Genotypes A, B, and C were rare: A in 3 cases (6.82%, 95% CI: 1.4–18.7), B in two cases (4.55%, 95% CI: 0.6–15.5), and C in one case (2.27%, 95% CI: 0.1–12.0). Subgenotyping assigned these to A2 (*n* = 3), B4 (*n* = 2), and C2 (*n* = 1), respectively. Thus, the overall subgenotype distribution among migrants was as follows: subgenotypes D1 and D2 were equally frequent (*n* = 16 each, 36.36%, 95% CI: 22.4–52.2), followed by D3 (*n* = 6, 13.64%, 95% CI: 5.2–27.4), A2 (*n* = 3, 6.82%, 95% CI: 1.4–18.7), B4 (*n* = 2, 4.55%, 95% CI: 0.6–15.5), and C2 (*n* = 1, 2.27%, 95% CI: 0.1–12.0).

### 2.5. Analysis of RT Region Amino Acid Substitutions

In the RT region of the HBV genome, amino acid substitutions were identified at 89 positions among the 44 detected isolates. Notably, a considerable proportion of these positions (36 positions) harbored substitutions that occurred only once, accounting for 2.3% (95% CI: 0.1–12.0). The highest frequencies of amino acid substitutions were observed at positions Y135S (substitutions in 26 cases, 59.1%, 95% CI: 43.2–73.7) and N248H (substitutions in 23 cases, 52.3%, 95% CI: 36.7–67.5).

Analysis of drug resistance mutations revealed their presence in five cases (11.4%, 95% CI: 3.8–24.6), all of which were infected with genotype D2. Specifically, the combination L180M + T184A + M204V was observed in three samples, and L180M + M204V in two. These mutations are known to confer resistance to lamivudine, entecavir, and telbivudine. One genotype C2 sample carried substitution T184L, which occurs at a position linked to entecavir resistance, but has not been previously reported as a resistance mutation. Finally, the HCC-related substitution M309K was detected in four samples (9.1%, 95% CI: 2.5–21.7). A complete list of all identified substitutions with their frequencies is presented in Appendix A Table A8.

### 2.6. Analysis of Amino Acid Substitutions in the MHR of the HBV Genome

**Figure 3:**
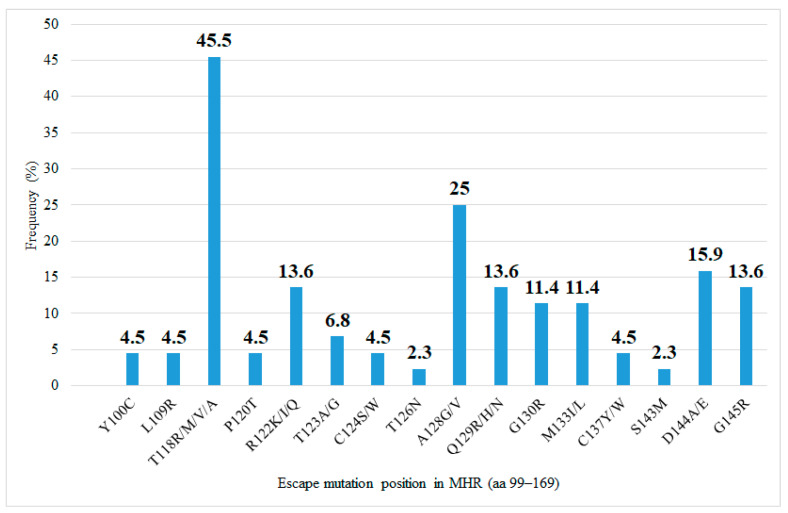
Frequency of escape mutations in the HBV MHR (aa 99–169).

Among the analyzed samples obtained from 44 migrants, amino acid substitutions within the major hydrophilic region (MHR, aa 99–169) were identified at 30 distinct positions. The highest substitution frequency was observed at position 127, with 27 substitutions, corresponding to 61.4% (95% CI: 45.5–75.6). A high frequency was also noted at position 118, where 20 substitutions were detected, accounting for 45.5% (95% CI: 30.4–61.2). Notably, the majority of substitutions were rare: ten distinct substitutions occurred only once each (2.3%, 95% CI: 0.1–12.0), and another ten substitutions occurred twice each (4.55%, 95% CI: 0.6–15.5). The prevalence of substitutions representing known immune escape mutations is illustrated in Figure 3. Detailed numerical data, including absolute counts, percentages, and 95% confidence intervals for all identified amino acid substitutions in the MHR, are provided in Appendix A Table A9.

Notably, escape mutations were primarily observed in individuals with OBI. Specifically, only two HBsAg-positive migrants harbored escape mutations, yielding a prevalence of 18.18% (95% CI: 2.28–51.78). By contrast, escape mutations were detected in 27 OBI patients, corresponding to 81.82% (95% CI: 64.54–93.02). This difference was statistically significant (Fisher’s exact test, *p* = 0.003).

### 2.7. Analysis of Mutations in the Precore/Core Region of the HBV Genome

In the BCP region, the double amino acid substitution A1762T/G1764A was detected in 11 samples (25.0%, 95% CI: 13.2–40.3). Among the 44 examined migrants, amino acid substitutions in the precore region were identified in only 26 individuals (59.09%, 95% CI: 43.2–73.7). Substitutions were detected at ten distinct positions. The absolute and relative frequencies of these substitutions, along with their 95% confidence intervals, are presented in Table 3.

**Figure 4:**
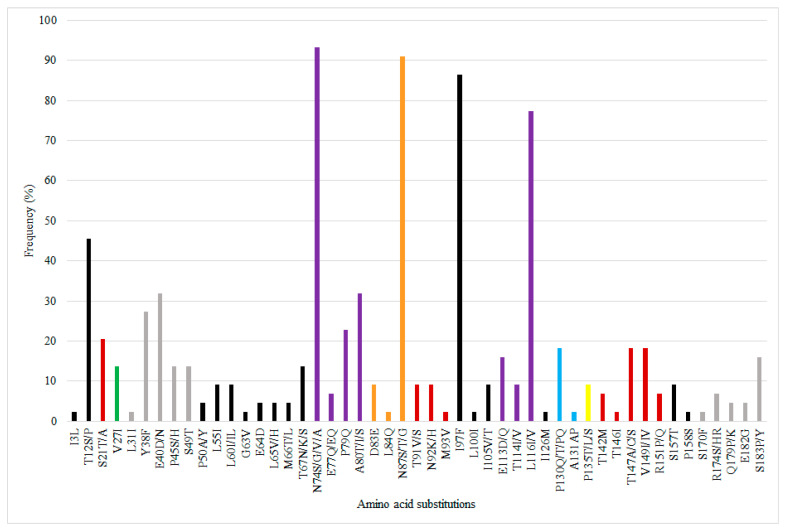
Distribution of core region amino acid substitutions across HBcAg immune epitopes. Colors denote epitope combinations: black, CD4+ T-cell epitopes only; green, CD8+ T-cell epitopes only; purple, B-cell epitopes only; red, CD4+ and CD8+ T-cell epitopes; orange, CD4+ T-cell and B-cell epitopes; yellow, CD8+ T-cell and B-cell epitopes; blue, all three epitope types; gray, positions with no known association with these epitopes.

In the core region, amino acid substitutions were identified at 49 distinct positions among the examined migrants. The highest substitution frequencies were observed at positions 74, 87, 97, and 116. Specifically, substitutions at position 74 were detected in 41 cases (93.18%, 95% CI: 81.3–98.6), followed by position 87 in 40 cases (90.91%, 95% CI: 78.3–97.5), position 97 in 38 cases (86.36%, 95% CI: 72.6–94.8), and position 116 in 34 cases (77.27%, 95% CI: 62.2–88.5). Across the core protein, several amino acid substitutions were situated within established HBcAg immune recognition domains. Specifically, substitutions involved CD4+ T-cell epitopes (aa 1–20, 50–69, 81–105, 117–131, 141–165), CD8+ T-cell epitopes (aa 18–27, 88–96, 130–140, 141–151), and B-cell epitopes (aa 74–89, 107–118, 127–138). Figure 4 maps these substitutions onto the corresponding epitope regions. The frequency data for all such substitutions are summarized in Appendix A Table A10.

## 3. Discussion

### 3.1. Overview of Principal Findings

In this molecular epidemiological study of 537 international migrants from 46 countries residing in St. Petersburg, Russia, we report several key findings with implications for both clinical practice and public health policy. First, the serological prevalence of HBsAg (2.61%) and anti-HBc (16.39%) confirms that migrants represent a population with significantly higher HBV burden compared to the general Russian population, where HBsAg prevalence is estimated at approximately 1–2% [32]. Second, we identified a strikingly high rate of OBI at 6.15% of the entire cohort and 6.31% among HBsAg-negative subjects, with the majority of OBI cases (54.5%) lacking any serological markers (anti-HBc or anti-HBs). Third, we documented extensive genetic diversity, with genotype D predominating (86.36%), but also the presence of genotypes A2, B4, and C2, reflecting the diverse origins of migrants. Fourth, we identified a significant accumulation of immune escape mutations in the MHR strongly associated with OBI (81.82% of OBI cases versus 18.18% of HBsAg-positive cases; *p* = 0.003). Fifth, we detected clinically relevant drug resistance mutations (L180M + M204V ± T184A) in 11.4% of isolates, all genotype D2, as well as precore stop codon W28* (31.82%) and basal core promoter A1762T/G1764A double substitution (25.0%). Collectively, these findings highlight the urgent need to include molecular HBV screening in mandatory medical examinations for arriving migrants.

### 3.2. Prevalence of HBsAg and Anti-HBc in the Migrant Cohort

The HBsAg prevalence of 2.61% (95% CI: 1.43–4.34) observed in our cohort aligns with previous reports on migrants in Russia, where rates of 2.8% have been described [32], and is comparable to findings from other European receiving countries. A systematic review of community-based screening among newly arrived migrants in Europe reported HBV prevalence up to 8.7% depending on region of origin [11]. In the EU/EEA, migrants accounted for 34% of all new HBV diagnoses in 2023 [10], underscoring the disproportionate burden in this population.

It is also worth noting that among the 14 HBsAg-positive individuals identified in this cohort, only 11 had detectable HBV DNA in plasma, despite the high sensitivity of our nested PCR assay and confirmation of HBsAg reactivity by neutralization testing. This pattern is well recognized in chronic hepatitis B and most plausibly reflects the production of HBsAg from HBV DNA sequences integrated into the host genome. This phenomenon occurs frequently during chronic infection, particularly in HBeAg-negative patients with low viremia, and does not require active viral replication [37]. Importantly, HBV integration has been associated with the persistence of HBsAg even after virological suppression and with an increased risk of hepatocellular carcinoma [37], underscoring the need for continued clinical surveillance even in HBsAg-positive individuals with undetectable viremia. This observation highlights the complementary value of molecular testing in accurately characterizing HBV infection status insofar as HBsAg positivity does not invariably indicate active viremia.

The anti-HBc prevalence of 16.39% indicates past or ongoing exposure, consistent with data from similar migrant cohorts in Europe [24,28]. Notably, we observed a marked age-related increase in anti-HBc prevalence, rising from 6.5% in individuals aged 18–29 years to 31.03% in those aged 40–49 years (χ^2^ = 28.41, *p* < 0.0001), reflecting cumulative exposure risk over time and possibly earlier vaccination policies in younger cohorts from certain countries of origin.

This age-dependent accumulation of anti-HBc is not unique to migrants. A recent population-based study in the same receiving region (Northwest Russia) reported a nearly identical pattern, with anti-HBc prevalence rising from 3.1% in children and adolescents to 26.4% in individuals aged ≥ 70 years [38]. The consistency of this age trend across both migrant and local populations suggests that cumulative exposure risk over a lifetime, rather than country-of-origin alone, is a major determinant of anti-HBc seropositivity.

The prevalence of anti-HBs IgG (26.82%, 95% CI: 23.11–30.78) in our cohort was comparable to estimates from other migrant populations in Europe, where pooled anti-HBs prevalence has been reported at 35.2% [39]. The serological pattern of “anti-HBs only” (18.06% of all migrants) most likely reflects vaccine-induced immunity, particularly among younger individuals who may have benefited from universal infant vaccination programs introduced in their countries of origin during the 1990s–2000s. However, in the absence of documented vaccination records, we cannot exclude the possibility that some of these cases represent past resolved infection with subsequent loss of anti-HBc reactivity over time. This phenomenon has been well described in long-term follow-up studies of hepatitis B [40,41].

### 3.3. Occult Hepatitis B Infection: A Hidden Reservoir

The OBI prevalence of 6.15% in the entire cohort and 6.31% among HBsAg-negative subjects is substantially higher than the global OBI prevalence of approximately 1–4% reported in general populations, but is comparable to rates observed in high-risk groups such as HIV-coinfected individuals, hemodialysis patients, and migrants from endemic areas [15,16,17]. A recent meta-analysis by Ji et al. reported a global OBI prevalence of 3.8% among HBsAg-negative individuals, with marked regional variation [15]. Our finding that the majority of OBI cases (54.5%) had no detectable anti-HBc or anti-HBs (so-called “seronegative OBI”) is particularly concerning from a public health perspective. These individuals would be entirely missed by all current serological screening protocols that rely on HBsAg and/or anti-HBc testing. Similar observations have been made in other migrant populations, although the proportion of seronegative OBI varies widely depending on the sensitivity of molecular methods and population characteristics [42]. The high proportion of seronegative OBI in our cohort is consistent with global estimates. A recent meta-analysis by Wu et al. (2024), which specifically examined HBsAg-negative/anti-HBc-negative populations across 21 studies, reported a pooled OBI prevalence of 3.0% in this seronegative subgroup [16]. These convergent findings across different populations (migrants, local residents) confirm that serological markers alone cannot reliably identify occult carriers, and that molecular testing is required even in the complete absence of any seropositivity. The uniformly low viral loads observed in OBI cases explain why standard PCR assays often fail to detect these infections, emphasizing the need for ultra-sensitive methods [13,14,43,44,45].

The high prevalence of OBI and escape mutations in migrants likely reflects a combination of factors: limited access to routine healthcare in countries of origin, absence of universal neonatal vaccination programs in older cohorts, and potential prior exposure to suboptimal antiviral therapy. Additionally, migration-associated stress and changes in immune status may facilitate reactivation of previously suppressed occult infections, though our cross-sectional design cannot address this directly.

### 3.4. Genetic Diversity and Importation of Non-Endemic HBV Genotypes

The predominance of genotype D (86.36%) in our cohort is consistent with the expected geographic distribution insofar as genotype D is the most prevalent genotype in Eastern Europe, the Mediterranean, and Central Asia [3,4,5,6]. The majority of our migrants originated from these aforementioned regions. Within genotype D, subgenotypes D1 and D2 were equally frequent (36.36% each), followed by D3 (13.64%). A detailed analysis of the country of origin revealed a distinct geographical segregation among D subgenotypes: subgenotype D1 was predominantly found in migrants from Central Asian countries (Uzbekistan, Tajikistan, Kyrgyzstan, Kazakhstan), whereas subgenotype D2 was almost exclusively detected in migrants from European countries (Belarus, Moldova, Ukraine, Serbia, Latvia). This distribution mirrors the well-documented phylogeographic separation of D1 (Central Asian/Middle Eastern lineage) and D2 (Eastern European lineage) and is consistent with previous Russian studies [46,47].

More importantly, we detected genotypes A2 (6.82%), B4 (4.55%), and C2 (2.27%). While genotype A2 is endemic to Russia, particularly in its European part, it is generally less prevalent than genotype D and is often associated with specific risk groups or regional pockets [47]. In contrast, genotypes B4 and C2 are not endemic to Russia; their presence in migrants from Vietnam (where these subgenotypes predominate) clearly reflects direct importation through migration. Genotype B4 is prevalent in Vietnam and Southeast Asia, while C2 is the dominant subgenotype in East Asia [6]. The importation of non-endemic genotypes into low-endemicity countries has been previously documented in Europe [8,9] and the United States [30] and may alter the local molecular epidemiology of HBV over time.

Certain genotypes are associated with different clinical outcomes. Genotype C is linked to higher rates of hepatocellular carcinoma (HCC) and delayed HBeAg seroconversion, while genotype A responds better to interferon-based therapy [5]. Clinicians in receiving countries should be aware of this diversity when managing chronic hepatitis B in migrant populations.

### 3.5. Immune Escape Mutations in the Major Hydrophilic Region as Drivers of OBI

The most striking finding of our study is the strong association between immune escape mutations in the MHR (amino acids 99–169) and OBI. Escape mutations were detected in 81.82% of OBI cases compared to only 18.18% of HBsAg-positive cases (*p* = 0.003). The most frequent substitutions were at positions T127P/I (61.4%) and T118R/M/V/A (45.5%). Both residues are located within the highly conserved “a” determinant (amino acids 124–147), a conformational epitope stabilized by four disulfide bonds (Cys-124, Cys-137, Cys-139, Cys-147). Substitutions at positions 118 and 127, particularly when involving proline (P127) or arginine (R118), disrupt the local secondary structure and alter the presentation of critical hydrophobic residues, thereby reducing the affinity of diagnostic anti-HBs antibodies by 10- to 100-fold [48,49]. Substitution G145R, a classic vaccine escape mutant found in 13.6% of our isolates, is known to abolish a key disulfide bond (Cys-147) and cause a conformational shift that completely eliminates binding of most monoclonal antibodies used in commercial HBsAg assays [13,27].

Our data support the current understanding that OBI often arises from mutations that alter HBsAg antigenicity, leading to false-negative results in ELISA-based screening [13,14]. Beyond diagnostic escape, the same conformational changes impair recognition by vaccine-induced anti-HBs antibodies. This has implications for post-exposure prophylaxis and vertical transmission prevention because hepatitis B immunoglobulin (HBIG) relies on neutralizing antibodies targeting the same “a” determinant epitopes. Variants carrying G145R or T127P/I have been shown to resist neutralization by HBIG, potentially compromising immunoprophylaxis in newborns from HBsAg-positive mothers [50,51].

The high frequency of escape mutations in our migrant cohort, particularly among those with OBI, suggests that these variants are not merely sporadic, but may be circulating at significant levels in the countries of origin. Similar findings have been reported among migrants from West Africa and Latin America [26,27]. Notably, the high frequency of escape mutations in genotype D isolates from our migrant cohort is consistent with recent reports documenting an “unexpected rise in the circulation of complex HBV variants enriched of HBsAg vaccine-escape mutations in HBV genotype-D” [52]. This suggests that the accumulation of MHR substitutions is not merely a random event, but may reflect a genotype-specific evolutionary adaptation under immune pressure.

From a public health perspective, the presence of vaccine escape mutants raises concerns about potential breakthrough infections in vaccinated individuals, although the clinical significance for transmission remains to be fully established [48,49]. The feminization of migration flows observed in our cohort (49.2% female, with a mean age of 40.1 years) and the high proportion of women of reproductive age (18–40 years) raises particular concerns regarding the risk of vertical transmission of HBV. This is especially relevant given the circulation of vaccine-escape mutants (e.g., G145R, T127P/I) that may compromise immunoprophylaxis with hepatitis B immunoglobulin (HBIG) [50,51]. Current screening protocols that rely solely on HBsAg would fail to detect occult infections in pregnant migrants, potentially leaving newborns unprotected. The inclusion of molecular HBV screening in routine antenatal care for migrant women from endemic regions should therefore be considered.

### 3.6. Drug Resistance Mutations in Treatment-Naive Migrants

Beyond transmission risks, clinically relevant drug resistance mutations were also detected in our cohort. We detected classic nucleos(t)ide analogue resistance mutations (L180M + M204V ± T184A) in 11.4% of isolates, all of which belonged to genotype D2. The L180M and M204V combination confers high-level resistance to lamivudine and telbivudine, and reduces susceptibility to entecavir (requiring additional mutations such as T184A, which we observed in three samples). Tenofovir remains fully active against these variants [28,29]. The presence of these mutations in treatment-naïve individuals is concerning as it suggests either transmission of resistant variants (primary resistance) or prior undocumented antiviral exposure in the countries of origin. Previous studies have documented the circulation of drug-resistant HBV variants in Central Asia and Eastern Europe, consistent with our observations [53,54,55]. For clinicians managing chronic hepatitis B in migrants, these findings underscore the importance of baseline resistance genotyping before initiating therapy, particularly for individuals originating from regions where suboptimal antiviral treatment may have been widely available without proper monitoring. One genotype C2 isolate carried the T184L substitution, which occurs at a position associated with entecavir resistance, but has not been previously confirmed as a resistance mutation; further phenotypic characterization would be required to determine its clinical significance.

### 3.7. Precore and Basal Core Promoter Mutations: Implications for HCC Risk

The detection of the precore stop codon mutation W28* in 31.82% of isolates and the BCP double substitution A1762T/G1764A in 25.0% has important clinical implications. The W28* mutation abolishes HBeAg production by introducing a premature stop codon, while the BCP mutations downregulate HBeAg expression at the transcriptional level [14]. Both mutations are associated with increased risk of HCC, particularly the BCP double mutation, which has been shown to upregulate viral replication and enhance the expression of the oncogenic HBx protein [21]. The co-occurrence of these mutations in a substantial proportion of our cohort, including among OBI cases, suggests that many of these migrants may be at elevated long-term risk for liver cirrhosis and HCC, even in the absence of detectable HBsAg. Longitudinal follow-up studies are needed to assess clinical outcomes in this population.

### 3.8. Core Region Immune Epitope Substitutions

We identified multiple amino acid substitutions within well-characterized CD4+ T-cell, CD8+ T-cell, and B-cell epitopes of the core protein (Figure 4). The highest substitution frequencies were observed at positions N74 (93.18%), N87 (90.91%), I97 (86.36%), and L116 (77.27%). N74 and N87 lie within the B-cell epitope 74–89, which is a major target of anti-HBc antibodies. Substitutions at these positions, particularly N74S and N87G, have been shown to reduce anti-HBc binding in enzyme immunoassays, potentially explaining the seronegative OBI cases we observed (54.5% with no detectable anti-HBc). I97F is located within the CD8+ T-cell epitope 88–96 (HLA-A2-restricted) and has been associated with impaired cytotoxic T-lymphocyte recognition and viral persistence. The accumulation of core mutations in our cohort may also affect the performance of anti-HBc immunoassays, although all our anti-HBc testing was based on IgG detection, which is less sensitive to single amino acid changes. Core protein mutations modulate both humoral and cellular immunity and contribute to chronicity, which is directly relevant to our findings [56,57,58].

### 3.9. Implications for Vertical Transmission

The feminization of migration flows observed in our cohort (49.2% female, with a mean age of 40.1 years) and the high proportion of women of reproductive age (18–40 years) raises particular concerns regarding the risk of vertical transmission of HBV. However, the evidence for mother-to-child transmission (MTCT) from mothers with OBI remains controversial [14,15,59], and the biological mechanisms underlying potential transmission are complex.

*Biological plausibility of OBI-related transmission.* Several lines of evidence support the biological plausibility of vertical transmission from mothers with OBI. First, the placental trophoblast layer represents the primary physical barrier between maternal and fetal circulations, and HBV must successfully cross this barrier to establish intrauterine infection. Wang et al. demonstrated that hepatitis B virus x protein (HBxAg) suppresses apoptosis and promotes the secretion of placental hormones (including human chorionic gonadotropin, progesterone, estrogen, and β-endorphin) in human placental trophoblasts via activation of the EGFR/Akt pathway [60]. This finding is particularly relevant to OBI as HBxAg is one of the few HBV proteins consistently expressed during occult infection, and its ability to modulate trophoblast survival and function may create a permissive environment for viral persistence at the maternal–fetal interface [60,61].

Second, Gao et al. showed that pregnant women with chronic HBV infection and high viral load exhibit an altered immune microenvironment characterized by increased proportions of effector/memory CD8+ T cells and upregulation of metallothionein-related genes and inflammatory pathways, suggesting that HBV infection during pregnancy is associated with a more active, rather than purely immunosuppressed, immune state [62]. This immune activation may have dual effects: it could facilitate viral control, but it may also contribute to inflammatory damage at the placental interface, potentially enhancing viral access to the fetal compartment. Third, the presence of immune escape mutations in the S gene (particularly within the “a” determinant) has been shown to alter HBsAg antigenicity and may contribute to HBsAg negativity in OBI cases [51,52]. These mutations may also affect the efficacy of HBIG and vaccine-induced antibodies, as discussed below.

*Direct evidence of vertical transmission from OBI-positive mothers.* Clinical evidence confirms that OBI can be transmitted perinatally. A case report from our group documented transmission from a mother with seronegative OBI (HBsAg-negative, anti-HBc-positive, HBV DNA-positive) to her infant, despite the child receiving HBV vaccination at birth. Both mother and child carried genotype D2 with identical S-region mutations (T114S, P127T) characteristic of OBI [63]. Furthermore, a prospective study by Eilard et al. found that approximately 10% of children born to HBeAg-positive mothers with high viral loads developed OBI despite adequate immunoprophylaxis with HBIG and vaccination [64]. A systematic review by Ji et al. estimated the global prevalence of OBI at 3.8% among HBsAg-negative individuals, with higher rates in high-risk populations, including those with a history of HBV exposure [15].

Studies from various regions have documented OBI prevalence among pregnant women ranging from 3.6% in Cameroon to 11.4% in Korea [65,66], highlighting substantial geographic variability and the need for region-specific screening strategies. In our own previous study of pregnant women in St. Petersburg, we found an OBI prevalence of 2.8% (38/1368) [1], which is considerably lower than the 6.15% observed in the present migrant cohort. This difference likely reflects the higher baseline HBV endemicity in the migrants’ countries of origin and underscores the added risk that migration poses for HBV importation, particularly among women of reproductive age. Additionally, a case report documented the de novo development of OBI during pregnancy, with HBV DNA levels peaking in the third trimester and returning to undetectable levels postpartum, suggesting that pregnancy-associated immunosuppression may facilitate the emergence of occult infection [67]. This observation implies that the risk of vertical transmission might be dynamic and potentially increase as pregnancy progresses.

*Escape mutations and neonatal prophylaxis.* Regarding the specific concern about escape mutations and neonatal prophylaxis, variants such as G145R and T127P/I have been shown to reduce anti-HBs binding and HBIG neutralization in vitro [48,51]. Lazarevic et al. documented that immune escape mutations are frequently associated with viral reactivation upon immunosuppression [51], while Piermatteo et al. recently reported an “unexpected rise in the circulation of complex HBV variants enriched of HBsAg vaccine-escape mutations in HBV genotype-D,” raising concerns about potential impacts on vaccination strategies [52]. However, the clinical impact of these mutations on the efficacy of HBIG plus vaccination in newborns remains uncertain. A review by Delghandi et al. concluded that despite proper active/passive immunoprophylaxis at birth, HBsAg-positive mothers may still transmit OBI to newborns, but emphasized that the risk is substantially reduced compared to the absence of prophylaxis [59]. Nevertheless, the high frequency of these variants in our migrant cohort warrants caution.

### 3.10. Public Health and Clinical Implications

Our findings have several practical implications. First, the current legal requirement for migrants in Russia (testing only for HIV, tuberculosis, leprosy, and syphilis, but not for viral hepatitis B or C) represents a critical regulatory gap [34]. Given the 2.61% HBsAg prevalence and 6.15% OBI prevalence we documented, mandatory HBsAg testing alone would miss the majority of HBV-infected individuals. We recommend that policymakers amend the mandatory medical examination for work permits to include at minimum HBsAg and anti-HBc, with confirmatory HBV DNA testing for those with isolated anti-HBc or seronegative OBI risk factors. Second, the high prevalence of immune escape mutations (81.82% in OBI) suggests that reliance on HBsAg testing for blood safety may be insufficient in migrant populations, although in Russia blood donors are screened by nucleic acid amplification testing, which mitigates this risk [23]. Third, clinicians should have a low threshold for HBV DNA testing in migrants from endemic regions, regardless of HBsAg status, especially before initiating immunosuppressive therapy or pregnancy, given the risk of reactivation [18,19,20]. Fourth, the presence of drug resistance mutations in treatment-naïve individuals argues for baseline genotypic resistance testing before starting antiviral therapy, as recommended by European and American guidelines.

### 3.11. Limitations of the Study

Several limitations of this study should be acknowledged. First, the cross-sectional design does not allow assessment of longitudinal outcomes such as viral reactivation or progression to hepatocellular carcinoma. Second, and most importantly, this was a purely serological and molecular epidemiological study; we did not collect any clinical data from the participants. Consequently, we lack information on liver function tests (ALT/AST), hepatic fibrosis staging, medical history (including prior antiviral treatment), or co-morbidities. The absence of these clinical parameters precludes us from correlating the identified viral genotypes and mutations (including OBI status, immune escape, and drug resistance variants) with specific clinical phenotypes, disease severity, or patient outcomes. Third, while we used ultracentrifugation to concentrate virus from plasma, viral loads in OBI cases were at the lower limit of detection; confirmation by digital PCR would provide more precise quantification. Fourth, the generalizability of our findings to other receiving countries may be limited by the specific composition of migrant flows to St. Petersburg, which are heavily weighted toward former Soviet republics. Fifth, we did not perform functional characterization of novel or rare mutations, which would be required to definitively establish their biological effects. Sixth, we lacked documented vaccination histories for participants, limiting our ability to distinguish vaccine-induced anti-HBs from that arising from resolved natural infection. This precludes definitive attribution of escape mutations to vaccine-driven selection pressure versus other mechanisms.

## 4. Materials and Methods

### 4.1. Study Design and Ethical Approval

The study material consisted of blood plasma samples obtained from 537 international migrants residing in the Russia’s Northwest Federal District (NWFD), collected during mandatory medical examination for obtaining a work permit at the Migration Department of the NWFD from February to December 2019. The study protocol was approved by the Local Ethics Committee of the St. Petersburg Pasteur Institute of Epidemiology and Microbiology (Protocol No. 67, dated 22 February 2017, and Protocol No. 97, dated 29 January 2020). All participants were informed about the purpose and methodology of the study and provided written informed consent. Data were collected using standardized face-to-face interviews with a formal questionnaire including basic socio-demographic characteristics (sex, age, country of origin).

### 4.2. Study Population

A total of 537 foreign nationals from 46 countries were enrolled in the study. Inclusion criteria were as follows: age ≥ 18 years, foreign citizenship, presentation for medical examination to obtain a work permit in the NWFD, and written informed consent to participate in the study and to provide a venous blood sample. Exclusion criteria were as follows: refusal to participate, inability to obtain a blood sample, or incomplete medical or demographic records. More than 80% of the examined individuals (84.2%) belonged to 11 out of 46 represented countries, including Ukraine (24.8%), Kazakhstan (14.2%), Uzbekistan (12.7%), Belarus (8.9%), Moldova (5.0%), Tajikistan (4.7%), Armenia (4.5%), Azerbaijan (2.8%), China (2.6%), Kyrgyzstan (2.0%), and Turkmenistan (2.0%). The overall study population consisted of 273 men (50.8%) and 264 women (49.2%). The age of the participants ranged from 18 to 90 years, with a mean age of 37.8 years (median 35 years). Among male patients, age ranged from 18 to 90 years, with a mean of 35.6 years. Among female patients, age ranged from 18 to 85 years, with a mean of 40.1 years. Participants were subdivided into the following age groups: 18–29 years (*n* = 200), 30–39 years (*n* = 138), 40–49 years (*n* = 87), 50–59 years (*n* = 56), and 60 years and older (*n* = 56).

### 4.3. Sample Transportation and Storage

Sample collection, plasma separation, virus concentration by ultracentrifugation, and transport conditions were performed as previously described [47]. The only difference was in aliquoting. For the present study, cryovials were prepared as follows for: ELISA (2 × 500 μL); PCR (2 × 250 μL and 1 × 500 μL); and sequencing (5000 μL).

### 4.4. Enzyme-Linked Immunosorbent Assay

Qualitative detection of HBsAg, anti-HBs IgG, and anti-HBcore IgG was carried out using the same ELISA protocol and commercial kits as described in our previous study [47]. Confirmatory neutralization testing for reactive or borderline HBsAg results was performed as described [47,68].

### 4.5. Nucleic Acid Extraction

DNA extraction was performed using the “NK-Magno-UltraPure-A” kit (Epitop, Saint Petersburg, Russia) according to the manufacturer’s instructions, as previously described [47]. However, the present study employed a modified multi-tiered strategy: initial screening from 200 μL plasma; confirmation of all HBV DNA-positive results by a second, blinded extraction from a fresh 200 μL aliquot; repeat extraction from 500 μL for inconclusive PCR cases (single-channel detection); and extraction from ≥5 mL for viral load quantification and sequencing. A negative control (sterile PBS) was included per extraction run.

### 4.6. HBV DNA Amplification

HBV DNA was amplified by nested PCR following Taormina Workshop guidelines for occult infection [14]. All samples were tested with an in-house nested PCR specifically adapted to detect concentrations as low as 5 IU/mL in 200 μL plasma, including HBsAg-negative specimens [43,44,45].

In the first amplification round, outer primers HBV longF/longR were used to generate a near-full-length genomic amplicon. The second round was a multiplex real-time reaction co-amplifying the HBV S-gene (FAM), X-gene (ROX), and human HPRT (Cy5, internal control). Primer and probe sequences for both rounds are summarized in Table 4. Thermal cycling conditions are provided in Supplementary Tables S1 and S2.

Positivity for HBV DNA required amplification in both the FAM and ROX channels, provided the Cy5 internal control was valid. Single-channel amplification was classified as inconclusive, prompting re-extraction from 500 μL plasma (see Section 2.5). Occult infection was defined by HBV DNA detection in two separate PCR runs using independently extracted aliquots, with no detectable HBsAg.

For quantitative assessment, a commercial PCR kit (Vector-Best, Novosibirsk, Russia, nominal sensitivity 29 IU/mL from 1 mL plasma) was used. DNA input was increased to 5 mL (Section 2.5), enhancing sensitivity, though the exact limit was not recalculated. Thus, values are reported as estimates. The same extract served for sequencing.

### 4.7. HBV Genomic Amplification and Sequencing

Overlapping fragments covering the complete HBV genome were amplified using previously published primer pairs [69,70,71,72,73]. PCR products were purified by ethanol precipitation according to a standard protocol. Purified DNA was quantified fluorometrically (Qubit 2.0, Thermo Fisher Scientific, Waltham, MA, USA) and assessed for integrity by agarose gel electrophoresis.

Sanger sequencing was performed using the ABI PRISM^®^ BigDye™ Terminator v3.1 Cycle Sequencing Kit (Applied Biosystems, Waltham, MA, USA). For each amplified fragment, both forward and reverse primers (1.6 pmol/μL each) were used in triplicate reactions, with 50–100 ng of template DNA per reaction. Sequencing products were purified by ethanol precipitation, dissolved in HI-DI™ formamide (Applied Biosystems, Waltham, MA, USA), and analyzed on an ABI Prism^®^ 3500 Genetic Analyzer (Applied Biosystems, Waltham, MA, USA) following manufacturer instructions.

### 4.8. Genotype and Mutation Analysis

Genotype and mutation analysis was performed as previously described [47]. Briefly, consensus sequences were assembled using Unipro UGENE v. 47 [74], aligned with ClustalW in MEGA v.11 [75], and phylogenetically analyzed by the neighbor-joining (NJ) method with 1000 bootstrap replicates. Evolutionary distances were computed using the Kimura 2-parameter (K2P) model. The Woolly Monkey HBV sequence (AY226578) was used as an outgroup to root the tree, and bootstrap values ≥ 70% were considered statistically supported. The nucleotide sequences obtained were submitted to the HBVdb (https://hbvdb.lyon.inserm.fr/HBVdb/HBVdbIndex (accessed on 14 March 2026)) [76], and Genafor (https://hbv.geno2pheno.org (accessed on 11 March 2026)) [77] databases to search for possible mutations. Amino acid sequences were deduced by translating the corresponding nucleotide sequences according to the open reading frame.

### 4.9. Statistical Analysis

Statistical data processing was carried out using the GraphPad Prism 5.0 (GraphPad Software, Inc., San Diego, CA, USA) software package. Key statistical results were independently verified using R software (version 4.3.2). Microsoft Excel (Microsoft Corp., Redmond, WA, USA) was used for data organization, table preparation, and graphical presentation. For all proportional estimates, 95% confidence intervals (CIs) were calculated using the exact Clopper-Pearson (beta-binomial) method, which provides valid interval estimates regardless of sample size, including small counts and proportions near the boundaries, without relying on normal approximation. For group comparisons, we used non-parametric tests appropriate to the data structure: Fisher’s exact test for 2 × 2 contingency tables and the Chi-square test with Yates’ continuity correction for larger tables. Correlation analysis was performed using Spearman’s rank correlation coefficient (rs). The regression lines shown in Figure A1 were fitted by the least-squares method for visualization purposes only and do not represent a separate regression analysis. Since all applied tests are distribution-free, no preliminary assessment of normality was required. A two-sided *p*-value < 0.05 was considered statistically significant.

## 5. Conclusions

This study shows that international migrants in St. Petersburg, Russia, carry a substantial burden of HBV infection, including a high prevalence of occult hepatitis B (6.15%), diverse genotypes (D1, D2, D3, A2, B4, C2), and clinically significant mutations. Immune escape variants were strongly associated with OBI (*p* = 0.003). Drug resistance mutations (11.4%) and HCC-associated precore/BCP mutations were also noted. The majority of OBI cases (54.5%) had no detectable serological markers, making them invisible to current screening protocols. These findings highlight an urgent need to revise mandatory medical examinations for arriving migrants to include molecular HBV detection. They also give context which can improve clinical management strategies in receiving countries.

## Figures and Tables

**Table 1:** Prevalence and distribution of hepatitis B serological markers (HBsAg, anti-HBc IgG, anti-HBs IgG) in the studied group.

Serological Marker (Prevalence)	abs., (*n*)	%	95% CI
HBsAg	14	2.61	1.43–4.34
Anti-HBs IgG	144	26.82	23.11–30.78
Anti-HBcore IgG	88	16.39	13.36–19.79
Seronegative	350	65.18	60.98–69.21
**Serological profile (distribution)**			
HBsAg	2	0.37	0.05–1.34
Anti-HBs IgG only	97	18.06	14.9–21.58
Anti-HBcore IgG only	29	5.4	3.65–7.66
HBsAg, anti-HBcore IgG	12	2.23	1.16–3.87
Anti-HBs IgG, anti-HBcore IgG	47	8.75	6.5–11.47

**Table 2:** Prevalence of HBV DNA among migrants by age group.

Age Group, Years	Number of Migrants	HBV DNA	
	*n*	abs., (*n*)	%, 95% CI
18–29	200	17	8.50 (5.03–13.26)
30–39	138	13	9.42 (5.11–15.57)
40–49	87	8	9.20 (4.05–17.32)
50–59	56	17	30.36 (18.78–44.10)
60+	56	2	3.57 (0.44–12.31)
Total	537	44	8.19 (6.02–10.04)

**Table 3:** Amino acid substitutions in the precore region of the HBV genome.

Amino Acid Substitution	Frequency of Occurrence in the Group (*n* = 44)	
	abs., *n*	%, 95% CI
L3R/F	8	18.18 (8.2–32.7)
C7S	5	11.36 (3.8–24.6)
S11F	1	2.27 (0.1–12.0)
T16I	1	2.27 (0.1–12.0)
V17F	5	11.36 (3.8–24.6)
A19G	1	2.27 (0.1–12.0)
G25R	5	11.36 (3.8–24.6)
W28S	1	2.27 (0.1–12.0)
W28*/*W	14	31.82 (18.6–47.6)
G29D/GD	15	34.09 (20.5–49.9)

**Table 4:** Oligonucleotide sequences used for nested PCR.

Round	Primer/Probe Name	Sequence (5′ → 3′)	Target
First	HBV longF	TTTTTCACCTCTGCCTAATCA	Full genome
First	HBV longR	AAAAAGTTGCATGRTGMTGG	Full genome
Second	HBV SrtF2	CACCTGTATTCCCATCCCATC	S gene
Second	HBV SrtR2	AGCCCTACGAACCACTGAACA	S gene
Second	HBV Srt-Z	FAM-AAACGGACTGAGGCCCACTCCCA-BHQ1	S gene probe
Second	HBV XrtF2	GTCTGTGCCTTCTCATCTGCC	X gene
Second	HBV XrtRd2	GTCGGTCGTTGACATTGCAG	X gene
Second	HBV Xrt-Z	ROX-TGTGCACTTCGCTTCACCTCTGC-BHQ2	X gene probe
Second	HPRT F	CTTGCTCGAGATGTGATGAAGG	HPRT
Second	HPRT R	CAGCAGGTCAGCAAAGAATTTATAG	HPRT
Second	HPRT-Z	Cy5-ATCACATTGTAGCCCTCTGTGTGCTCAAGG-BHQ2	HPRT probe

## Data Availability

The original contributions presented in this study are included in the article/Supplementary Material. Further inquiries can be directed to the corresponding author.

## Supplementary Materials

The following supporting information can be downloaded at: https://www.mdpi.com/article/10.3390/ijms27136065/s1.

## Appendix A

**Table A1:** Prevalence of serological markers by sex.

Serological Marker (Prevalence)	Male (*n* = 273)			Female (*n* = 264)		
	abs., (*n*)	%	95% CI	abs., (*n*)	%	95% CI
HBsAg	9	3.3	1.52–6.17	5	1.89	0.62–4.38
Anti-HBs IgG	69	25.27	20.25–30.9	75	28.41	23.03–34.26
Anti-HBcore IgG	45	16.48	12.3–21.45	43	16.29	12.05–21.32

**Table A2:** Prevalence of HBsAg, anti-HBc IgG, and anti-HBs IgG by age group.

Age Group, Years	*n*	HBsAg			Anti-HBcore IgG			Anti-HBs IgG		
		abs., (*n*)	%	95% CI	abs., (*n*)	%	95% CI	abs., (*n*)	%	95% CI
18–29	200	5	2.5	0.82–5.74	13	6.5	3.51–10.86	57	28.5	22.36–35.29
30–39	138	4	2.9	0.80–7.26	21	15.22	9.67–22.32	32	23.19	16.43–31.13
40–49	87	3	3.45	0.72–9.75	27	31.03	21.55–41.86	27	31.03	21.55–41.86
50–59	56	2	3.57	0.44–12.31	13	23.21	12.98–36.42	14	25	14.39–38.37
60+	56	0	0	0–6.38	14	25	14.39–38.37	14	25	14.39–38.37

**Table A3:** Country-specific contribution to HBsAg seropositivity among migrants (*n* = 14).

Country	*n*	%	95% CI
Armenia	1	7.14	0.18–33.87
Belarus	1	7.14	0.18–33.87
Kyrgyzstan	1	7.14	0.18–33.87
Moldova	3	21.43	4.66–50.80
Tajikistan	3	21.43	4.66–50.80
Turkmenistan	1	7.14	0.18–33.87
Uzbekistan	4	28.57	8.39–58.10

**Table A4:** Country-specific contribution to anti-HBc IgG seropositivity among migrants (*n* = 144).

Country	*n*	%	95% CI
Azerbaijan	6	4.17	1.54–8.85
United Kingdom	3	2.08	0.43–5.97
Armenia	3	2.08	0.43–5.97
Afghanistan	1	0.69	0.02–3.81
Belarus	13	9.03	4.89–14.94
Bulgaria	1	0.69	0.02–3.81
Vietnam	1	0.69	0.02–3.81
Egypt	1	0.69	0.02–3.81
India	2	1.39	0.17–4.93
Iran	2	1.39	0.17–4.93
Kazakhstan	26	18.06	12.15–25.33
Cameroon	1	0.69	0.02–3.81
Kyrgyzstan	5	3.47	1.14–7.92
China	4	2.78	0.76–6.96
Korea	1	0.69	0.02–3.81
Lebanon	1	0.69	0.02–3.81
Lithuania	1	0.69	0.02–3.81
Mexico	1	0.69	0.02–3.81
Moldova	9	6.25	2.90–11.53
Pakistan	1	0.69	0.02–3.81
Serbia	2	1.39	0.17–4.93
Syria	1	0.69	0.02–3.81
USA	2	1.39	0.17–4.93
Tajikistan	8	5.56	2.43–10.65
Turkmenistan	1	0.69	0.02–3.81
Uzbekistan	26	18.06	12.15–25.33
Ukraine	19	13.19	8.13–19.84
Estonia	2	1.39	0.17–4.93

**Table A5:** Country-specific contribution to anti-HBs IgG seropositivity among migrants (*n* = 88).

Country	*n*	%	95% CI
Azerbaijan	4	4.55	1.25–11.23
Algeria	1	1.14	0.03–6.17
Armenia	1	1.14	0.03–6.17
Belarus	3	3.41	0.71–9.64
Bulgaria	1	1.14	0.03–6.17
United Kingdom	1	1.14	0.03–6.17
Vietnam	1	1.14	0.03–6.17
Kazakhstan	6	6.82	2.54–14.25
Cameroon	1	1.14	0.03–6.17
Kyrgyzstan	4	4.55	1.25–11.23
China	1	1.14	0.03–6.17
Korea	1	1.14	0.03–6.17
Latvia	2	2.27	0.28–7.97
Moldova	8	9.09	4.01–17.13
Serbia	4	4.55	1.25–11.23
Tajikistan	10	11.36	5.59–19.91
Turkmenistan	1	1.14	0.03–6.17
Uzbekistan	19	21.59	13.53–31.65
Ukraine	19	21.59	13.53–31.65

**Table A6:** Country-specific contribution to HBV DNA positivity among migrants (*n* = 44).

Country	*n*	%	95% CI
Armenia	1	2.27	0.06–12.02
Belarus	6	13.64	5.17–27.35
Vietnam	3	6.82	1.43–18.66
Kazakhstan	5	11.36	3.79–24.56
Kyrgyzstan	1	2.27	0.06–12.02
China	2	4.55	0.56–15.47
Latvia	1	2.27	0.06–12.02
Lithuania	1	2.27	0.06–12.02
Moldova	3	6.82	1.43–18.66
Serbia	1	2.27	0.06–12.02
Tajikistan	3	6.82	1.43–18.66
Turkmenistan	2	4.55	0.56–15.47
Uzbekistan	11	25	13.19–40.34
Ukraine	4	9.09	2.53–21.67

**Table A7:** Country-specific prevalence of HBV DNA among migrants (countries with at least one positive case).

Country	*n*	HBV DNA + (*n*)	%, 95% CI
Armenia	24	1	4.17 (0.11–21.12)
Belarus	48	6	12.50 (4.73–25.25)
Vietnam	4	3	75.00 (19.41–99.37)
Kazakhstan	76	5	6.58 (2.17–14.69)
Kyrgyzstan	11	1	9.09 (0.23–41.28)
China	14	2	14.29 (1.78–42.81)
Latvia	9	1	11.11 (0.28–48.25)
Lithuania	3	1	33.33 (0.84–90.57)
Moldova	27	3	11.11 (2.35–29.16)
Serbia	7	1	14.29 (0.36–57.87)
Tajikistan	25	3	12.00 (2.55–31.22)
Turkmenistan	11	2	18.18 (2.28–51.78)
Uzbekistan	68	11	16.18 (8.36–27.10)
Ukraine	133	4	3.01 (0.83–7.52)

**Table A8:** Frequency of amino acid substitutions in the RT region of HBV isolates (*n* = 44).

Amino Acid Substitution	Frequency of Occurrence in the Group (*n* = 44)			
	abs., *n*	%	95% CI −	95% CI +
A7T/D	5	11.4	3.8	24.6
H9Y	1	2.3	0.1	12
N13H	2	4.5	0.6	15.5
I16T	2	4.5	0.6	15.5
R18K	2	4.5	0.6	15.5
T19A	1	2.3	0.1	12
P20L	1	2.3	0.1	12
A21S	6	13.6	5.2	27.4
T24S	1	2.3	0.1	12
T37S	1	2.3	0.1	12
A38E	5	11.4	3.8	24.6
I/N53V/S/E	3	6.8	1.4	18.7
N53D	1	2.3	0.1	12
Y54H/S/N	11	25	13.2	40.3
V63G	1	2.3	0.1	12
L77V	1	2.3	0.1	12
A87G	1	2.3	0.1	12
Y89L	1	2.3	0.1	12
L91I/T	3	6.8	1.4	18.7
H94L	1	2.3	0.1	12
V103A/I	16	36.4	22.4	52.2
S106T/A	3	6.8	1.4	18.7
P109S	4	9.1	2.5	21.7
R110G	2	4.5	0.6	15.5
V112F/L	2	4.5	0.6	15.5
L115V/M	4	9.1	2.5	21.7
N118T	2	4.5	0.6	15.5
F122L	6	13.6	5.2	27.4
N124H/Y	4	9.1	2.5	21.7
H124Y/Q	4	9.1	2.5	21.7
H126P/R	19	43.2	28.3	59
T128N	2	4.5	0.6	15.5
M129L/LM	4	9.1	2.5	21.7
Q130P	3	6.8	1.4	18.7
N131S/R	3	6.8	1.4	18.7
L132M	2	4.5	0.6	15.5
N/D134D/E/N	5	11.4	3.8	24.6
Y135S	26	59.1	43.2	73.7
C136W	1	2.3	0.1	12
S137Q	1	2.3	0.1	12
R138W/T	5	11.4	3.8	24.6
Q139E	1	2.3	0.1	12
Y141S	1	2.3	0.1	12
V142I/L	4	9.1	2.5	21.7
M145V	1	2.3	0.1	12
L146V	1	2.3	0.1	12
Y148D	1	2.3	0.1	12
Q149K	2	4.5	0.6	15.5
Y151C	1	2.3	0.1	12
R153Q/G/K	8	18.2	8.2	32.7
L157M	1	2.3	0.1	12
Y158C	1	2.3	0.1	12
L164M	3	6.8	1.4	18.7
L180M	5	11.4	3.8	24.6
T184L	1	2.3	0.1	12
T184A	3	6.8	1.4	18.7
H197P	2	4.5	0.6	15.5
M204V	5	11.4	3.8	24.6
D206H	1	2.3	0.1	12
V207M	1	2.3	0.1	12
S213ST	1	2.3	0.1	12
L217R	3	6.8	1.4	18.7
S219A	1	2.3	0.1	12
S223A	1	2.3	0.1	12
L229F	5	11.4	3.8	24.6
L231V	1	2.3	0.1	12
N238T	3	6.8	1.4	18.7
K241R	2	4.5	0.6	15.5
Y245H	1	2.3	0.1	12
N248H	23	52.3	36.7	67.5
F249N	1	2.3	0.1	12
T259S	2	4.5	0.6	15.5
D263E	7	15.9	6.6	30.1
I266R	8	18.2	8.2	32.7
Q267H	2	4.5	0.6	15.5
K268*K	1	2.3	0.1	12
M/E271L/D	8	18.2	8.2	32.7
V278I	7	15.9	6.6	30.1
W284C	1	2.3	0.1	12
V291A	1	2.3	0.1	12
L293H	4	9.1	2.5	21.7
Y305D	1	2.3	0.1	12
M309K	4	9.1	2.5	21.7
P310L	4	9.1	2.5	21.7
Q316H	1	2.3	0.1	12
A317S	1	2.3	0.1	12
K318R	7	15.9	6.6	30.1
S/C332R/Y	4	9.1	2.5	21.7
K333Q/N	3	6.8	1.4	18.7

**Table A9:** Absolute and relative frequencies of all amino acid substitutions identified in the major hydrophilic region (MHR, aa 99–169) of the HBV genome.

Amino Acid Substitution	Frequency of Occurrence in the Group (*n* = 44)			
	abs., *n*	%	95% CI −	95% CI +
Y100C	2	4.5	0.6	15.5
Q101H	2	4.5	0.6	15.5
M103I	2	4.5	0.6	15.5
V106A	1	2.3	0.1	12
L109R/P	2	4.5	0.6	15.5
I110L	2	4.5	0.6	15.5
G112E	2	4.5	0.6	15.5
T116N	1	2.3	0.1	12
T118R/M/V/A	20	45.5	30.4	61.2
P120T	2	4.5	0.6	15.5
R122K/I/Q	6	13.6	5.2	27.4
T123A/G	3	6.8	1.4	18.7
C124S/W	2	4.5	0.6	15.5
T125M	2	4.5	0.6	15.5
I126N	1	2.3	0.1	12
T127P/I	27	61.4	45.5	75.6
A128G/V	11	25	13.2	40.3
Q129R/H/N	6	13.6	5.2	27.4
G130R	5	11.4	3.8	24.6
M133I/L	5	11.4	3.8	24.6
C137Y/W	2	4.5	0.6	15.5
C139W	1	2.3	0.1	12
T140I	1	2.3	0.1	12
S143M	1	2.3	0.1	12
D144A/E	7	15.9	6.6	30.1
G145R	6	13.6	5.2	27.4
I150V	1	2.3	0.1	12
G159A	1	2.3	0.1	12
K160R	1	2.3	0.1	12
S167L	1	2.3	0.1	12

**Table A10:** Amino acid substitutions identified in the core region of the study group: absolute and relative frequencies with 95% confidence intervals.

Amino Acid Substitution	Frequency of Occurrence in the Group (*n* = 44)			
	abs., *n*	%	95% CI −	95% CI +
I3L	1	2.27	0.1	12
T12S/P	20	45.45	30.4	61.2
S21T/A	9	20.45	9.8	35.3
V27I	6	13.64	5.2	27.4
L31I	1	2.27	0.1	12
Y38F	12	27.27	15	42.8
E40D/N	14	31.82	18.6	47.6
P45S/H	6	13.64	5.2	27.4
S49T	6	13.64	5.2	27.4
P50A/Y	2	4.55	0.6	15.5
L55I	4	9.09	2.5	21.7
L60I/IL	4	9.09	2.5	21.7
G63V	1	2.27	0.1	12
E64D	2	4.55	0.6	15.5
L65V/H	2	4.55	0.6	15.5
M66T/L	2	4.55	0.6	15.5
T67N/K/S	6	13.64	5.2	27.4
N74S/G/V/A	41	93.18	81.3	98.6
E77Q/EQ	3	6.82	1.4	18.7
P79Q	10	22.73	11.5	37.8
A80T/I/S	14	31.82	18.6	47.6
D83E	4	9.09	2.5	21.7
L84Q	1	2.27	0.1	12
N87S/T/G	40	90.91	78.3	97.5
T91V/S	4	9.09	2.5	21.7
N92K/H	4	9.09	2.5	21.7
M93V	1	2.27	0.1	12
I97F	38	86.36	72.6	94.8
L100I	1	2.27	0.1	12
I105V/T	4	9.09	2.5	21.7
E113D/Q	7	15.91	6.6	30.1
T114I/V	4	9.09	2.5	21.7
L116I/V	34	77.27	62.2	88.5
I126M	1	2.27	0.1	12
P130Q/T/PQ	8	18.18	8.2	32.7
A131AP	1	2.27	0.1	12
P135T/L/S	4	9.09	2.5	21.7
T142M	3	6.82	1.4	18.7
T146I	1	2.27	0.1	12
T147A/C/S	8	18.18	8.2	32.7
V149I/IV	8	18.18	8.2	32.7
R151P/Q	3	6.82	1.4	18.7
S157T	4	9.09	2.5	21.7
P158S	1	2.27	0.1	12
S170F	1	2.27	0.1	12
R174S/HR	3	6.82	1.4	18.7
Q179P/K	2	4.55	0.6	15.5
E182G	2	4.55	0.6	15.5
S183P/Y	7	15.91	6.6	30.1
