# Supplementary material for: Genetic Diversity, Occult Hepatitis B, and Mutational Signatures in Migrants from Regions with Varying HBV Endemicity: Importation of Diverse Viral Variants into St. Petersburg, Russia
Source: Int J Mol Sci. 2026 Jul 6;27(13):6065. doi: 10.3390/ijms27136065 (PMC13361592; doi:10.3390/ijms27136065)
Supplement: Supplementary file 1 [file ijms-27-06065-s001.zip › ijms-4401168-supplementary.pdf]

Supplementary Table S1. Thermal cycling conditions for the first round of nested PCR.

**Block**	**Step**	**Temperature (°C)**	**Time**	**Number of cycles**
1	Initial denaturation	95	5 min	1
2	Denaturation	95	20 sec	20
	Annealing	52	30 sec	
	Extension	72	3 min 50 sec	
3	Final extension	72	7 min	1
4	Denaturation	95	20 sec	20
	Annealing	52	30 sec	
	Extension	72	3 min 50 sec	
5	Final extension	72	7 min	1
6	Denaturation	95	20 sec	10
	Annealing	50	30 sec	
	Extension	72	3 min 50 sec	
7	Final extension	72	7 min	1
8	Hold	4	∞	–

Supplementary Table S2. Thermal cycling conditions for the second round of nested PCR.

**Block**	**Step**	**Temperature (°C)**	**Time**	**Fluorescence reading**	**Number of cycles**
1	Initial denaturation	95	5 min	–	1
2	Denaturation	95	10 s	–	7
	Annealing	50	20 s	–	
	Extension	72	40 s	–	
3	Final extension	72	5 min	–	1
4	Denaturation	95	10 s	–	45
	Annealing	58	20 s	FAM, ROX, CY5	
	Extension	72	40 s	–	
5	Hold	4	∞	–	–
